# 

**Published:** 1841-04-01

**Authors:** 


### BIBLIOGRAPHICAL RECORD.

1. Illustrations of Cutaneous Disease:—a series of delineations, &c. &c. By Robert Willis, M.D. &c. Fasciculus 22. Jan. 1841.

2. A popular Essay on the Value and Present Condition of the Practice of Vaccination, &c. By a Physician. Octavo, pp. 32. Birmingham. 1838.

3. History of Embalming, and of Preparations in Anatomy, Pathology, and Natural History; including an Account of a New Process for Embalming. By G. N. Gannal. Paris, 1838. Translated with notes and additions. By R. Harlan, M.D. 8vo. pp. 264. Philadelphia, 1840.

4. Phrenological Journal, and Magazine of moral Science. Quarterly. No. 66. Jan. 1, 1841. Maclachlan and Stewart.

5. Magdalenism. An Enquiry into the Extent, Causes, and Consequences of Prostitution in Edinburgh. By W. Tait, Surgeon. Octavo, pp. 266. Edinburgh, P. Rickard, 1841.

6. Retrospect of Practical Medicine and Surgery. Being a half-yearly Journal, containing a retrospective view of every discovery and practical improvement in the Medical Sciences. By W. Braithewaite, Esq. No. 2, July and December, 1840. Octavo, pp. 416. Simpkin and Co.

☞ *This work increases in interest and utility.*

7. The Anatomy of the Arteries of the Human Body, &c. Part IV. By Richard Quain, Esq. &c. Taylor and Walton, Jan. 1841.

8. Medicinischer Argos. Herausgegeben, Von den D. D. Hacker, und Prof. Hohl. Leipzig, 1840. Nos. 1 and 5, Vol. 1, p. 800.

☞ *We have noticed this new and interesting periodical elsewhere.*

9. On the Principles of Sound; their application to the New Houses of Parliament, and assimilation with the Mechanism of the Ear. By A. W. Webster, author of a Treatise on the Structure of the Ear, and Deafness. 8vo. pp. 112. Simpkin and Marshall, 1841.

10. A Letter to the Right Hon. Viscount Melbourne, with the outlines of a Bill regulating the Practice of Surgeons, Apothecaries, and Chemists and Druggists, &c. By Martin Sinclair, M.D. Senior Medical Officer to the Hulme Dispensary. 8vo. pp. 36. Highley, 1841.

11. Nuces Philosophicæ: or the Philosophy of Things, as developed from the study of the Philosophy of Words. By Edward Johnson, Esq. author of "Life, Health, and Disease." No. I. Jan. 1841. Simpkin and Co. Price one shilling.

12. The Anatomy of the Nerves of the Uterus. By Robert Lee, M.D. F.R.S. Physician to the British Lying-in Hospital, and Lecturer on Midwifery at St. George's Hospital. Folio, with two Plates. Balliere, Regent-street, Jan. 1841.

13. The Cause and Treatment of Curvature of the Spine, and Diseases of the Vertebral Column. Illustrated with cases and plates. By E. W. Tuson, F.R.S. F.L.S. Surgeon to the Middlesex Hospital, &c. &c. Octavo, pp. 282. Churchill, 1841.

14. The Domestic Management of the Sick-room, necessary in aid of Medical Treatment, for the cure of Diseases. By Ant. Todd Thomson, M.D. &c. Octavo, pp. 506, with numerous wood-cuts. Longman and Co. 1841.

☞ *This excellent hand-maid to the medical practitioner should be in every family—especially where there are children or invalids.*

15. The Second Annual Report of the Northampton General Lunatic Asylum, (opened August 1st, 1838), from July 1839, to June 1840—together with an earnest appeal to the public on its behalf. Wellingborough, 1841.

☞ *This Report is from the pen of Mr. T. O. Prichard, and does him credit.*

16. Edinburgh Monthly Journal of Medical Science. Edited by Dr. J. R. Cormack, M.D. Nos. 1 and 3, Jan. and Feb. 1841.—*In exchange.*

---

17. A Practical Treatise on the Causes, Nature, and Treatment of Strictures of the Urethra; with a Review of the Different Modes of Treatment. Illustrated by cases. By Francis Burdett Courtney, Member of the Royal College of Surgeons. Octavo, pp. 244. Simpkin and Marshall, 1841.

---

18. Egypt and Mohammed Ali. Illustrative of the condition of his Slaves and Subjects, &c. By R. R. Madden, M. D. &c. Octavo. Hamilton, Adams, and Co. 1841.

---

19. Provincial Medical and Surgical Journal, in weekly numbers, received regularly. —*In exchange.*

---

20. Report of the Superintendent of the Boston Lunatic Hospital, and Physician of the Public Institutions at South Boston. July 1, 1840.

☞ *A sensible and able Report.*

---

21. A few Hints addressed to Medical Students about to visit the Parisian Hospitals. By a Physician. Duodecimo, pp. 56. Churchill, 1841.

☞ *A useful little vade mecum.*

---

22. The Surgical Anatomy of Inguinal Herniæ, the Testis, and its Coverings. By Thomas Morton, one of the Demonstrators of Anatomy in University College, London, &c. illustrated with lithographic plates and wood engravings. Royal 8vo. pp. 123. Taylor and Walton. 1841.

---

23. The Library of Medicine. Arranged and Edited by Alex. Tweedie, M.D. F.R.S. &c. Vol. VI. Midwifery, by Edward Rigby, M.D. Physician to the General Lying-in Hospital, &c. pp. 314. Whittaker 1841.

---

24. On Paralytic, Neuralgic, and other Nervous Diseases of the Eye. By Arthur Jacob, M.D. Professor, &c. (From the Dublin Medical Press), 1841.

---

25. Seventh Annual Report of the State Lunatic Hospital, at Worcester. Boston, 1840.

26. The Anatomy of the Arteries. By Richard Quain, Professor of Anatomy to University College. Part 5, five plates, with letter-press, price 12s. Jan. 1841.

---

27. A Series of Anatomical Sketches and Diagrams, with Descriptions and References. By Thomas Wormald, Assistant-Surgeon, &c. Bartholomew's Hospital—and Andrew Melville McWhinnie, Teacher of Practical Anatomy in the same. Part IV. price 4s. S. Highley, 1841.

---

28. Annales de la Chirurgie, Francaise, et Etrangere. Par Begin, Marchall, Velpeau, Vidal, &c. No. I. Jan. 1841. Bailliere.—*In exchange.*

---

29. An Atlas of Plates illustrative of the Principles and Practice of Obstetric Medicine and Surgery, with descriptive letter-press. By Francis Ramsbotham, M.D. &c. Nos. 13 and 14, price 1s. 6d. Churchill, Jan. and Feb. 1841.

☞ *This concludes the work.*

---

30. Illustrations of Cutaneous Diseases, &c. &c. By Robert Willis, M.D. Fasciculus V. Bailliere, 1841.

---

31. A Practical Treatise on the Venereal Disease, &c. By F. C. Skey, Esq. F.R.S. Octavo, pp. 195. Churchill, 1841.

---

32. Resolutions of the Gloucestershire Medical Association, held February 16th, 1841.

---

33. Petition to Parliament from the Gloucestershire Medical Association, respecting the Poor Laws.

---

34. First Report of the Progress of Legal Education in Ireland, upon the principle of the Dublin Law Institute, to the Council of the Society, with Suggestions, &c. Dublin: Hodges and Co. Dec. 1840.

---

35. The Principal Baths of Germany, considered with reference to their Remedial Efficacy, &c. &c. By Edwin Lee, Esq. M.R.C.S. Part the Second—Central and Southern Germany. Small 8vo. pp. 134. Whittaker, 1841,

---

36. The Mineral Springs of England, and their Curative Efficacy, with remarks on bathing, &c. By Edwin Lee, Esq. Whittaker, 1841. Small 8vo. pp. 124.

